# Surgery Management of Rare Hypertrophic Frenum in an Infant

**DOI:** 10.1155/2014/168192

**Published:** 2014-08-10

**Authors:** Sheila de Carvalho Stroppa, Juliana Yassue Barbosa da Silva, Maria Cristina Reis Tavares, João Gilberto Duda, Estela Maris Losso

**Affiliations:** ^1^Pediatric Dentistry, Positivo University, Curitiba, PR, Brazil; ^2^Federal University of Pernambuco, Avenida Professor Moraes Rego, No. 1235, Cidade Universitária, 50670-901 Recife, PE, Brazil; ^3^Post-Graduation Program, Positivo University, PR, Brazil; ^4^Masters Program in Clinical Dentistry, Pediatric Dentistry, Positivo University, Rua Pedro Viriato Parigot de Souza, No. 5300, Campo Comprido, 81280-330 Curitiba, PR, Brazil

## Abstract

To report a rare case of a lateral frenum hypertrophy in an infant, this paper describes the case of a girl who came to a first dental appointment when she was 4 months old. A hypertrophic lateral frenum in the upper left canine region was detected. A great depression in the gingival rodet separated the anterior maxillary segment from the posterior one and also decreased the lip mobility in this region. A frenectomy was performed when the patient was 11 months old and the clinical follow-up was done up to the age of 30 months. There was normalization in the vestibular insertion of the lateral frenum, lip mobility, physiological development of the maxilla, and eruption of the upper incisors, canines, and first primary molars. Infants should go to a dental examination precociously in order to detect possible congenital and development alterations.

## 1. Introduction

A baby's first dental visit should be done before 1 year of age to promote oral health. Sometimes, however, babies have intraoral development alterations that require an early diagnosis so clinical planning can be made [1], thus preventing the possible repercussions for the development of the oral cavity.

The baby's mouth has anatomical details that are age characteristic, and the dental professional must know this anatomy to be able to diagnose nonphysiological alterations. The labial frenum is composed of two layers of epithelia that enclose loose vascular connective tissue. It is considered anomalous when an increased volume is present, causing undesirable clinical conditions, decreased lip mobility, and speech disorders [2].

The scientific literature reports a 16–25% prevalence of an abnormal upper labial frenum in newborns, but no case reports or surveys on the prevalence of a lateral frenum in babies have been found [3, 4].

The aim of this paper is to report a case of an infant's hypertrophic lateral frenum from the moment of diagnosis until 19 months after surgery, including clinical follow-up during this process.

## 2. Case Report

A female child with leukoderma, 4 months old, and in good health, arrived for dental care at the Baby Clinic of Positivo University. The child's medical history revealed no prenatal or postnatal problems or any history of oral trauma. The child was exclusively breastfed, without any sucking difficulties and no pacifier use. She had no alterations in the extraoral region.

**Figure 1:**
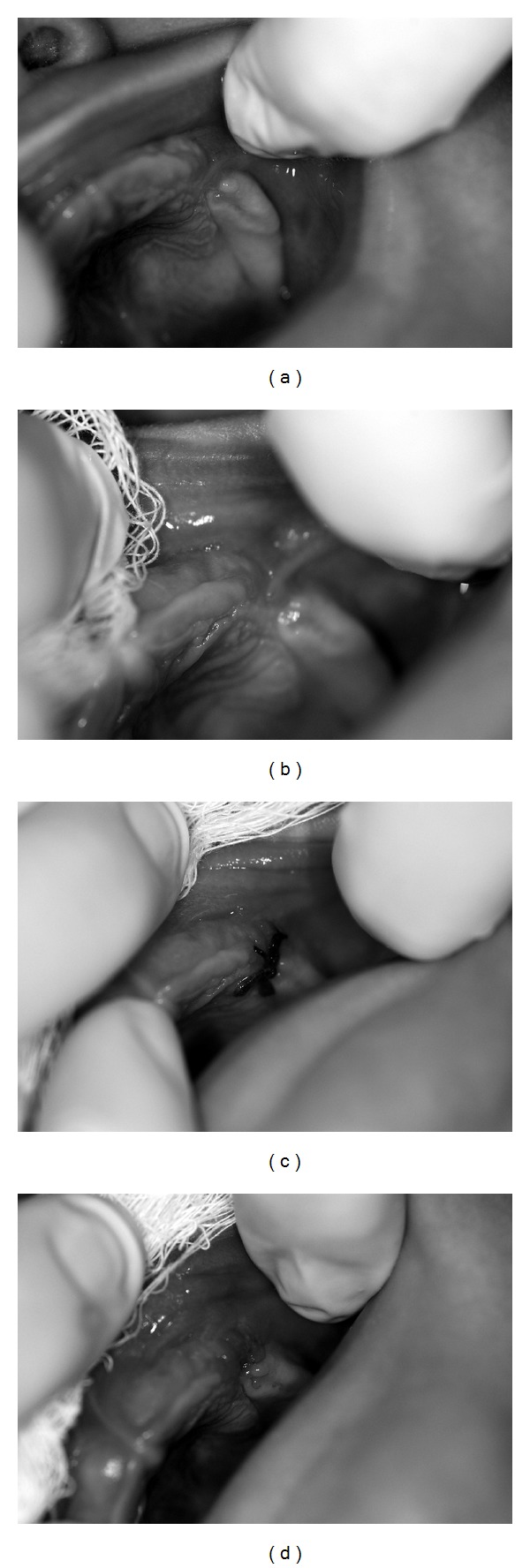
(a) Initial clinical aspects of left upper region at 4 months of age, (b) presurgical clinical aspects at 11 months of age, (c) seven days after surgery, before suture removal, and (d) seven days after surgery, after suture removal.

The intraoral examination revealed a hypertrophic upper left vestibular lateral frenum with palatal insertion in the upper left canine region with a depression in the gingival rodet that separates the anterior maxillary segment from the posterior maxillary segment and the labial mobility lack in the region (Figure 1(a)). The other structures had a normal appearance. Appointments were held every 2 months and with no change in the lateral frenum insertion (Figure 1(b)). Therefore, a frenectomy was performed when she was 11 months old. Seven days after surgery (Figure 1(c)), the sutures were removed (Figure 1(d)).

**Figure 2:**
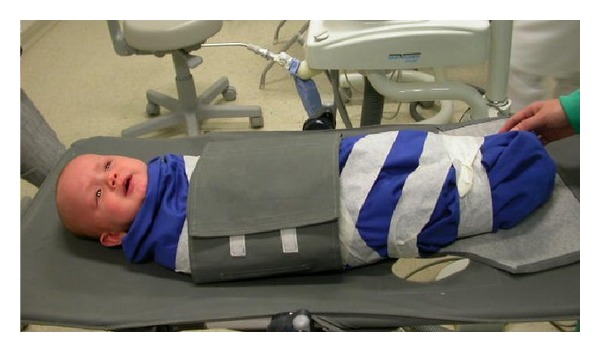
Physical restraint utilized for surgical procedure.

On the day of the surgery, the child was in good general condition and the surgery was performed at Positivo University Baby Clinic. The surgical procedure was conducted using physical restraint and without sedation (Figure 2). Topical anesthesia has been performed with EMLA (Astra Zeneca) with the aid of dry gauze for 3 minutes and the area was anaesthetized with local infiltration by using 2% mepivacaine (1 : 100,000 adrenaline). The surgery was performed with a scalpel blade (number 15) and 2 incisions were made in the lateral frenum, forming a wedge-shaped piece that was removed, followed by removal of fibers attached to the bone and sutured. The suture was removed after seven days.

**Figure 3:**
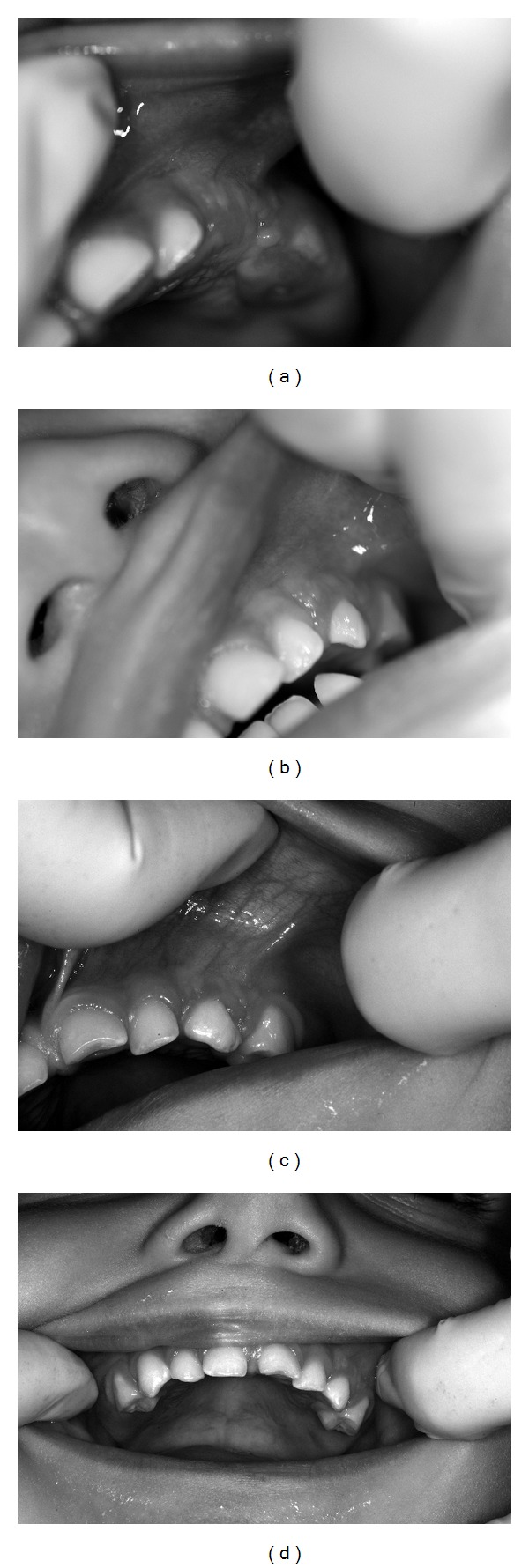
(a) Clinical aspects 7 months after surgery at 18 months of age, (b) clinical aspects 13 months after surgery at 24 months of age, (c) clinical aspects 19 months after surgery, left upper region, at 30 months of age, and (d) clinical aspects 19 months after surgery, anterior view, at 30 months of age.

A clinical followup was performed every 2 months to evaluate the insertion of the frenum and the eruption of the deciduous teeth.  Figure 3(a) shows the second intraoral clinical aspect 7 months after surgery when the child was 18 months old. The presence of the deciduous upper left first molar and visible anatomical improvement of the upper arch was observed. At the 13-month postsurgical clinical followup (Figure 3(b)), the physiological development of the maxilla, the eruption of the upper left canine and left first primary molar, the frenum vestibular insertion, and lip mobility were normalized. Nineteen months after surgery, at 30 months of age, the child showed tooth eruption and normality in the region (Figures 3(c) and 3(d)).

## 3. Discussion

The labial frenum is considered pathological if it is abnormally large or wide and if no attached gingiva is apparent along the midline [5]. In the present case report, we observed a wide lateral frenum with insertion into the gingival border that caused anterior and posterior segmentation of the alveolar crest.

A tomography was not requested because the cost was too high for the family and sedation would be required. Difficulty was also encountered in taking periapical X-rays because of the patient's age. Therefore, clinical monitoring of the case was chosen, with simultaneous orientation toward the prevention of dental caries and malocclusion. Clinical followup was performed every 2 months to verify the position of the lateral frenum insertion. Because no improvement in the insertion was observed and to maintain the segmentation of the alveolar crest, surgery was performed at 11 months of age before the eruption of the upper incisors to avoid alveolar bone loss and the persistence of morphological gingival border alterations. The clinical followup after surgery showed improvement of the insertion of the lateral frenum, better morphology of the arch, and eruption of the upper left canine and left first primary molar in their correct locations.

In conclusion, babies must have their first dental appointment early in life so that possible diagnoses of congenital and development alterations can be made as soon as possible. This will allow an individualized plan to prevent future problems in bone development and malocclusion.
